# Patient Preferences for Artificial Intelligence in Medical Imaging: A Single-Center Cross-Sectional Survey

**DOI:** 10.1007/s10278-025-01629-w

**Published:** 2025-08-07

**Authors:** Kennedye N. McGhee, D. Jonah Barrett, Omar Safarini, Asser Abou Elkassem, John T. Eddins, Andrew D. Smith, Steven A. Rothenberg

**Affiliations:** 1 University of Alabama at Birmingham Marnix. E. Heersink School of Medicine, 619 19th Street South, Birmingham, AL 35233 USA; 2 Department of Radiology, St. Jude Children’s Research Hospital, 262 Danny Thomas Place, Memphis, TN USA

**Keywords:** Artificial Intelligence, Patient preferences, Medical imaging, Transparency, Informed consent, Radiology practice

## Abstract

Artificial Intelligence (AI) is rapidly being implemented into clinical practice to improve diagnostic accuracy and reduce provider burnout. However, patient self-perceived knowledge and perceptions of AI’s role in their care remain unclear. This study aims to explore patient preferences regarding the use of and communication of AI in their care for patients undergoing cross-sectional imaging exams. This single-center cross-sectional study, a structured questionnaire recruited patients undergoing outpatient CT or MRI examinations between June and July 2024 to assess baseline self-perceived knowledge of AI, perspectives on AI in clinical care, preferences regarding AI-generated results, and economic considerations related to AI, using Likert scales and categorical questions. A total of 226 participants (143 females; mean age 53 years) were surveyed with 67.4% (151/224) reporting having minimal to no knowledge of AI in medicine, with lower knowledge levels associated with lower socioeconomic status (*p* < .001). 90.3% (204/226) believed they should be informed about the use of AI in their care, and 91.1% (204/224) supported the right to opt out. Additionally, 91.1% (204/224) of participants expressed a strong preference for being informed when AI was involved in interpreting their medical images. 65.6% (143/218) indicated that they would not accept a screening imaging exam exclusively interpreted by an AI algorithm. Finally, 91.1% (204/224) of participants wanted disclosure when AI was used and 89.1% (196/220) felt such disclosure and clarification of discrepancies should be considered standard care. To align AI adoption with patient preferences and expectations, radiology practices must prioritize disclosure, patient engagement, and standardized documentation of AI use without being overly burdensome to the diagnostic workflow. Patients prefer transparency for AI utilization in their care, and our study highlights the discrepancy between patient preferences and current clinical practice. Patients are not expected to determine the technical aspects of an image examination such as acquisition parameters or reconstruction kernel and must trust their providers to act in their best interest. Clear communication of how AI is being used in their care should be provided in ways that do not overly burden the radiologist.

## Introduction

Artificial Intelligence (AI) is being rapidly adopted in medical imaging to enhance diagnostic performance and workflow efficiency to help address the increasing imaging volumes and a shortage of radiologists [1]. The number of FDA-cleared AI devices continues to grow, with 1012 products approved, including 777 specific to radiology, according to the FDA Artificial Intelligence and Machine Learning (AI/ML)-Enabled Medical Devices database [2]. While the utilization of AI applications continues to grow, it is not well known how patients perceive the use of this technology in their diagnostic care.

Recent studies that are not specific to radiology indicate that patients prioritize being informed about AI use in their medical care [3, 4] and often desire the option to opt out [5]. Many patients prefer seeking care at facilities incorporating AI into their workflow [6] and desire opportunities to be engaged in AI development while ensuring diverse populations are included [7]. Additionally, most patients support AI technologies that can provide earlier diagnoses and more accurate treatments [8]. With regard to medical imaging, patients value proof of AI’s efficacy, knowledge of the imaging process, and how accountability is handled [9, 10]. Many patients hope AI will improve access, reduce wait times, and increase diagnostic accuracy [11]. Nevertheless, patients prefer a trained, human physician to AI-powered machines in diagnosis and interpretation of results and prefer that AI be used as a second reader [12, 13]. Patients also value the personal interaction and transparency provided by human radiologists [14].

Several studies have explored how sociodemographic factors influence trust and acceptance of AI in healthcare. Patients with higher education and prior AI knowledge tend to place greater trust in its diagnostic capabilities and treatment planning [15, 16]. Age is also a factor, as older patients appear significantly less likely to approve of AI for medical decision-making [17, 18]. Gender differences emerge in some studies, with males on average showing more pro-AI attitudes than females [19, 20]. The influence of race and ethnicity varies, with some studies finding race affects AI acceptance [17], while others report no significant group differences [20, 21]. Limited research on socioeconomic status (SES) suggests higher SES is linked to greater interest in healthcare AI [22] and more comfort with AI-assisted care among those from higher median-income backgrounds [23]. Many patients believe AI can reduce healthcare disparities by improving access [11, 24], while others worry it may worsen inequities by benefiting those already advantaged [12, 25].

Ensuring that AI utilization aligns with patient expectations is crucial for maintaining trust and autonomy in medical decision-making. A potential gap exists in how patients comprehend the use of AI’s role in their imaging and patient preferences. Understanding these concerns can guide healthcare providers and policymakers in improving communication strategies and fostering patient confidence and transparency in AI-assisted diagnostics. However, patient awareness of AI’s role in radiology, their preferences for its communication, and their understanding of its implications remain largely unexplored. The objective of this study is to explore patient preferences regarding the use of and communication of AI in their care for patients undergoing cross-sectional imaging exams.

## Materials and Methods

### Study Design and Patient Selection

This observational, cross-sectional, HIPAA-compliant, single-center study was approved by the UAB institutional review board (IRB-300012224). Individuals undergoing outpatient Computed Tomography (CT) and Magnetic Resonance Imaging (MRI) for any indication were recruited by a trained medical student (K.M.) between June 2024 and July 2024. Before enrollment, all patients were provided with a comprehensive description of the study, including potential risks and benefits, after which written informed consent was obtained. Individuals who declined participation were not recorded, precluding calculation of a response rate. Patients then completed a self-administered survey using an iPad (Apple Inc., California) prior to their imaging exam. The recruiting team member was available to answer any questions about the survey. Patients were not required to answer any question that they did not feel comfortable answering.

### Survey Description

The survey was designed by a second-year medical student (K.M.) with feedback from two board-certified radiologists (S.R., A.S.) and implemented in REDCap [26, 27]. It constituted a series of 5-point Likert scale questions and single-best-answer questions aimed at assessing patients’ self-perceived knowledge, understanding, and preferences on the use of AI in medical imaging. The questionnaire comprised a total of 23 questions divided into four sections: (a) demographics, (b) patient perspectives on AI use in clinical care, (c) patient perspectives on receiving AI results, (d) patient perspectives on the economics of AI. The survey questions used can be found in Table 1.

- The first section gathered demographic information such as age, gender, race and ethnicity, educational attainment, and household income, aiming to understand how they might influence patient attitudes and preferences regarding AI in medical imaging.
- The second section assessed patient perspectives on the use of AI in clinical care, including patients’ self-perceived knowledge and comfort levels regarding AI in medicine, preferences for being informed when AI is used, willingness to receive AI-driven care, and opinions on the right to opt out of AI-assisted medical practices.
- The third section focused on patient perspectives on receiving AI-generated results, exploring whether patients wanted to receive AI interpretations of their medical images alongside those of their radiologists and how they preferred discrepancies between AI and radiologist interpretations to be addressed.
- The final section addressed patient views on the economics of AI, specifically regarding the financial aspects of AI in medical imaging, assessing patients’ willingness to pay for AI-enhanced diagnostic services and their expectations regarding insurance coverage for such technologies.

**Table 1:** Study survey items

Item	Content
1	What level of knowledge do you have of AI* in medicine?
2	When an FDA-cleared AI algorithm is used to enhance clinical care, do you believe medical centers should inform their patients about the use of this technology?
3	I prefer to go to an imaging center that uses AI algorithms
4	Should patients have the right to opt out of using medical AI, even if the medical doctors believe it is helpful?
5	If there is strong evidence that AI improves clinical care, I would want AI applied to my medical images
6	If AI can detect diseases in my images that would otherwise be missed, I would want this kind of AI used for my care
7	If AI was applied to my medical images, I should be informed that AI was used
8	If AI was applied to my medical images, I should be informed of the result
9	If a doctor used AI to assist with the interpretation of your medical images and the doctor disagrees with the AI result, how should the doctor inform the patient in their report? Select the best single answer
10	If a doctor used AI to assist with interpretation of your medical images, which of the following should be standard of care?
11	If your doctor disagrees with the AI results for your medical images, how should he/she proceed with your care?
12	If a doctor used AI to assist with interpretation of your medical images, how should the results be provided to you?
13	At an academic medical center, my data and images may be used for research and quality improvement that does not directly impact my care. My radiology images should be used for AI research
14	I would pay an additional fee for AI to check my images for various diseases that could be missed by a doctor
15	If AI could detect 10 additional serious diseases that my doctor could miss, my medical insurance should pay for this type of service
16	I would accept a screening imaging exam that is exclusively assessed by an AI algorithm without a radiologist interpretation (e.g. no human oversight)
17	If your medical insurance would NOT pay for AI to detect additional serious diseases that your doctor could miss, what is the maximum amount that you would pay out of pocket for this service?

### Statistical Analysis

This survey was exploratory and a power analysis was not performed. Counts and frequencies were calculated for each survey question. Chi-square goodness-of-fit tests were performed to evaluate whether the observed frequencies of age, gender, education, and annual household income demographics deviated from the expected frequencies under the assumption of equal distribution across the groups. A subgroup analysis on the effect of socioeconomic status (SES) was performed in tertiles based on income range using thresholds from the United States Census Bureau and the Pew Research Center: low SES (< $30,000), middle SES ($30,000 – $120,000), and high SES (> $120,000) [28, 29]. Kruskal–Wallis tests were conducted to determine the effects of SES on patients’ responses to the Likert-scale items, followed by Dunn’s post hoc tests. Chi-square tests were used to analyze the effects of these demographic variables on categorical items. Fisher’s exact tests were used for categorical values. *p* < 0.05 was considered statistically significant. Statistical analysis was conducted using R Statistical Software (v4.3.2; R Core Team 2023) by (J.J.).

## Results

### Patients Characteristics

**Fig. 1:**
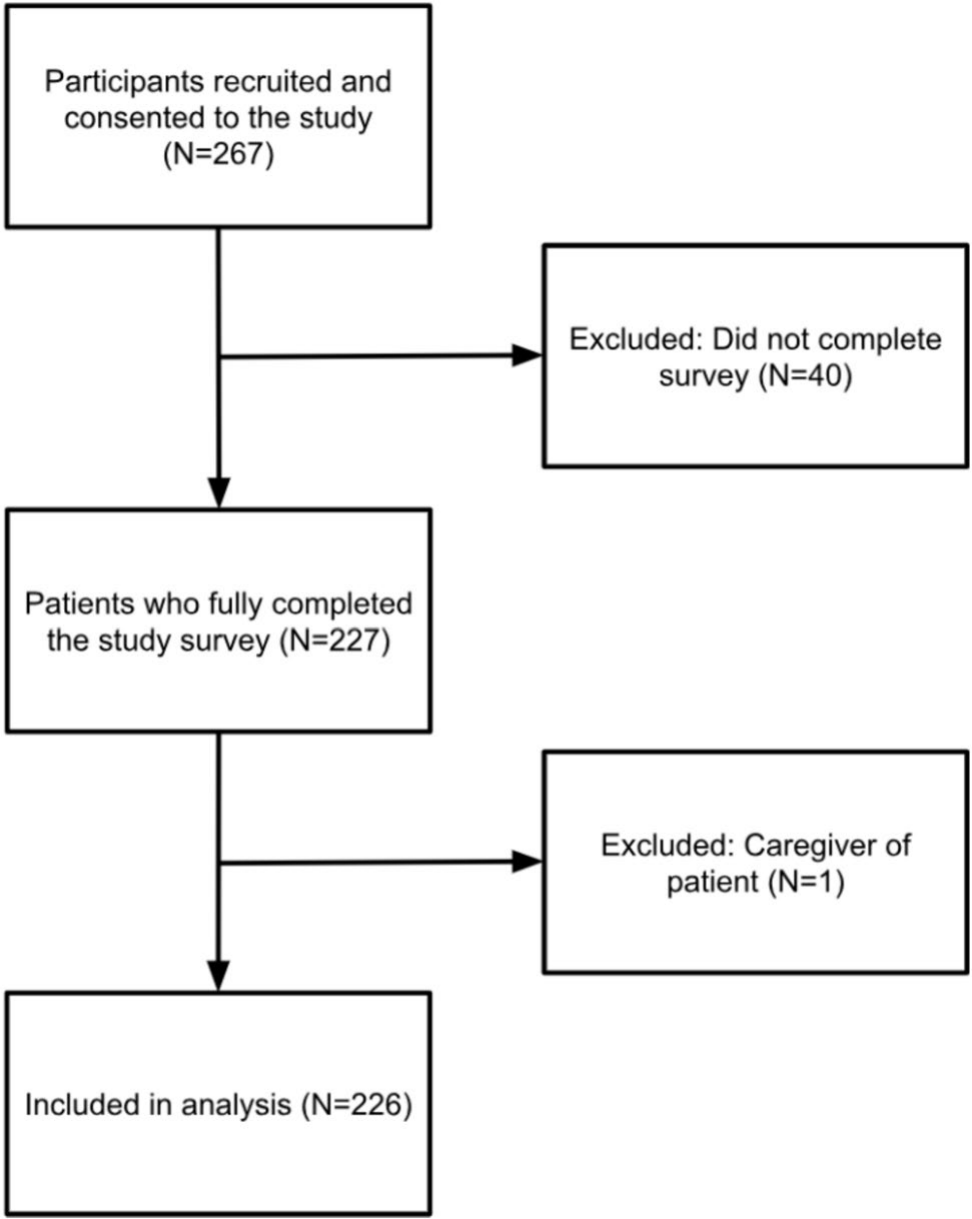
Flow chart of study patients

Of 267 participants recruited and consented to the study, 226 were included in the final analysis. Forty participants were excluded for not finishing the survey, and one participant was excluded because they were later determined to be a caregiver rather than a patient. The number of individuals who were approached and asked to participate but declined was not recorded. Overall study flow is detailed in Fig. 1. Responses were not mandatory for each survey item, resulting in varying numbers of responses across the different survey items. The participants were predominantly female (63.3%, 143/226) compared to males (35.8%, 81/226). During the recruitment period (June–August 2024), 17,422 outpatient MR and CT examinations were performed at our facility, with 9775 (56.1%) on female patients and 7632 (43.9%). Most participants were middle-aged to older adults, with 42.5% (96/226) between 51 and 70 years of age and 30.1% (68/226) between 31 and 50 years, while younger adults (18–30 years) and elderly patients (71 or older) represented smaller proportions at 8.8% (20/226) and 15.9% (36/226), respectively. The study cohort was predominantly White (69.5%, 157/226), with Black or African American participants representing the second largest group (21.7%, 49/226). Most participants had some college or trade/technical training (27.0%, 61/226), a college degree (30.1%, 68/226), or an advanced degree (20.4%, 46/226). Regarding socioeconomic status, nearly half of the patients (46.9%, 105/226) were middle SES, while high SES and low SES groups represented 22.8% (51/226) and 15.6% (35/226) of the cohort, respectively. Additional description of participants’ demographic information is detailed in Table 2.

**Table 2:** Demographic characteristics of participants

*Characteristics*	*Total (n* = *226)*
Age*	
18–30	20 (8.8%)
31–50	68 (30.1%)
51–70	96 (42.5%)
71 or older	36 (15.9%)
Prefer not to answer	2 (0.9%)
Gender*	
Male	81 (35.8%)
Female	143 (63.3%)
Prefer not to answer	2 (0.9%)
Race	
White	157 (69.5%)
Black or African American	49 (21.7%)
American Indian or Alaska Native	1 (0.4%)
Asian	3 (1.3%)
Other/Prefer not to answer	15 (6.6%)
Education*	
Less than a high school diploma	9 (4.0%)
High school diploma or equivalent (e.g., GED)	39 (17.3%)
Some college or trade/technical/vocational training	61 (27.0%)
College degree (Associate or bachelor’s degree)	68 (30.1%)
Advanced degree (Master’s or doctorate)	46 (20.4%)
Prefer not to answer	1 (0.4%)
Socioeconomic status (SES)	
Low SES (Under $30,000)	35 (15.6%)
Middle SES ($30,001 – $120,000)	105 (46.9%)
High SES (Greater than $120,000)	51 (22.8%)
Prefer not to answer	33 (14.7%)

^*^Chi-square goodness-of-fit tests, p ≤ 0.05; GED – General Equivalency Diploma; values are reported as number of patients with percentages in parentheses

### Self-Perceived Knowledge of AI

**Fig. 2:**
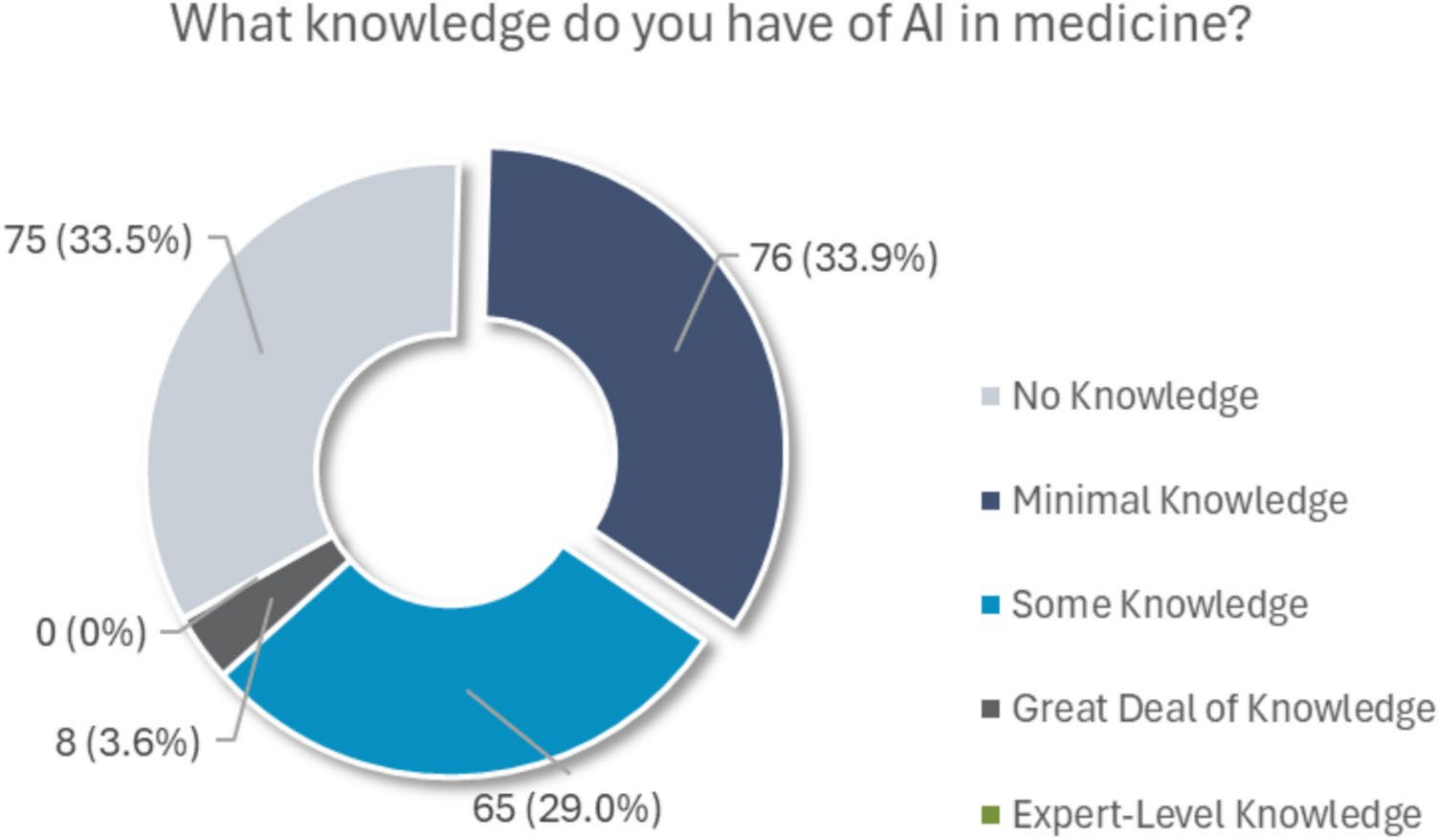
Pie chart of participants’ responses to the question, “What knowledge do you have of AI in medicine?”

Full details of participants’ responses to survey items can be found in Table 3, along with the p-values of the statistical tests examining the association between SES and participant responses. The majority (67.4%,151/224) of participants reported having minimal to no knowledge of AI in medicine. 29.0% (65/224) reported having some knowledge, and 3.6% (8/224) reported having a great deal of knowledge. The spectrum of self-perceived AI knowledge among participants is depicted in Fig. 2. Participants’ self-perceived level of AI knowledge was associated with their SES (Chi-square *p* < 0.001), with individuals in the high SES group claiming more AI knowledge (54.9% reporting some/great deal of knowledge) than the middle SES group (29.1%; Fisher’s exact *p* < 0.001) and low SES group (17.1%; *p* < 0.001).

**Table 3:** Participants’ responses to study items

*Item*	*Strongly disagree*		*Disagree*	*Neutral*	*Agree*	*Strongly agree*	SES (p-value)*
I prefer to go to an imaging center that uses AI algorithms. (*n* = 223)	11 (4.9%)		21 (9.4%)	140 (62.8%)	40 (17.9%)	11 (4.9%)	.606
If there is strong evidence that AI improves clinical care, I would want AI applied to my medical images. (*n* = 226)	7 (3.1%)		2 (0.9%)	56 (24.8%)	102 (45.1%)	59 (26.1%)	.005
If AI can detect diseases in my images that would otherwise be missed, I would want this kind of AI used for my care. (*n* = 225)	3 (1.3%)		3 (1.3%)	25 (11.1%)	101 (44.9%)	93 (41.3%)	.003
If AI was applied to my medical images, I should be informed that AI was used. (*n* = 226)	2 (0.9%)		1 (0.4%)	19 (8.4%)	65 (28.8%)	139 (61.5%)	.077
If AI was applied to my medical images, I should be informed of the result. (*n* = 224)	2 (0.9%)		1 (0.4%)	17 (7.6%)	59 (26.3%)	145 (64.7%)	.013
At an academic medical center, my data and images may be used for research and quality improvement that does not directly impact my care. My radiology images should be used for AI research. (*n* = 222)	11 (5.0%)		14 (6.3%)	71 (32.0%)	82 (36.9%)	44 (19.8%)	.022
I would pay an additional fee for AI to check my images for various diseases that could be missed by a doctor. (*n* = 222)	21 (9.5%)		41 (18.5%)	79 (35.6%)	59 (26.6%)	22 (9.9%)	.015
If AI could detect 10 additional serious diseases that my doctor could miss, my medical insurance should pay for this type of service. (n = 218)	4 (1.8%)		6 (2.8%)	22 (10.1%)	68 (31.2%)	118 (54.1%)	.020
I would accept a screening imaging exam that is exclusively assessed by an AI algorithm without a radiologist interpretation (e.g., no human oversight). (*n* = 218)	60 (27.5%)		83 (38.1%)	42 (19.3%)	21 (9.6%)	12 (5.5%)	.008
*Item*		*Choices/count (%)*					SES (p-value)*
What level of knowledge do you have of AI in medicine? (*n* = 224)		No knowledge — 75 (33.5%); Minimal knowledge — 76 (33.9%); Some knowledge — 65 (29.0%); A great deal of knowledge — 8 (3.6%); Expert-level knowledge — 0 (0%)					<.001
When an FDA-cleared AI algorithm is used to enhance clinical care, do you believe medical centers should inform their patients about the use of this technology? (*n* = 223)		Yes, patients deserve to know — 199 (89.2%); No, informing is not necessary — 8 (3.6%).; Unsure/I don’t know — 16 (7.2%)					.543
Should patients have the right to opt out of using medical AI, even if the medical doctors believe it is helpful? (*n* = 224)		Yes, patients deserve the right to opt-out — 204 (91.1%); No, patients don’t need the right to opt-out — 2 (0.9%); Unsure/I don’t know — 18 (8.0%)					.542
If a doctor used AI to assist with the interpretation of your medical images and the doctor disagrees with the AI result, how should the doctor inform the patient in their report? Select the best single answer. (*n* = 222)		The doctor should provide an overall summary without providing information on the AI result — 19 (8.6%); The doctor should provide an overall summary and explain why their interpretation disagrees with the AI result — 201 (90.5%); Other — 2 (0.9%)					.024
If a doctor used AI to assist with interpretation of your medical images, which of the following should be standard of care? (*n* = 220)		The doctor should not inform that AI was used and hide the AI result — 2 (0.9%); The doctor should inform that AI was used but hide the AI result — 15 (6.8%); The doctor should inform that AI was used and hide the AI result if he/she disagrees with the interpretation — 8 (3.6%); The doctor should inform that AI was used and provide the AI result, explaining any difference in interpretation — 196 (89.1%); e. Other — 2 (0.9%)					.073
If your doctor disagrees with the AI results for your medical images, how should he/she proceed with your care? (*n* = 219)		The doctor should use the AI result and their best judgment but hide the AI result — 21 (9.5%); The doctor should use their interpretation of images and AI result, without directly informing you of the AI result — 88 (40.0%); Other — 110 (50.0%)					.198
If a doctor used AI to assist with interpretation of your medical images, how should the results be provided to you? (*n* = 216)		The doctor should provide an interpretation without displaying the AI results (whether or not they agree) — 19 (8.8%); The doctor should use the AI, but only provide their final interpretation without the AI result shown — 11 (5.1%); The doctor should provide both their interpretation and the AI results (whether or not they agree) — 186 (86.1%)					.107
If your medical insurance would NOT pay for AI to detect additional serious diseases that your doctor could miss, what is the maximum amount that you would pay out of pocket for this service? (*n* = 220)		$0 per CT scan — 59 (26.8%); $10 per CT scan — 34 (15.0%); $20 per CT scan — 36 (16.4%); $50 per CT scan — 50 (22.7%); $100 per CT scan — 41 (18.6%)					.002

^*^p-values of Kruskal–Wallis (first 9 items) and chi-square tests (last 8 items) examining the association between SES and patients’ responses to survey items, *SES* socioeconomic status

### Patient Perspectives on AI Use in Clinical Care

**Fig. 3:**
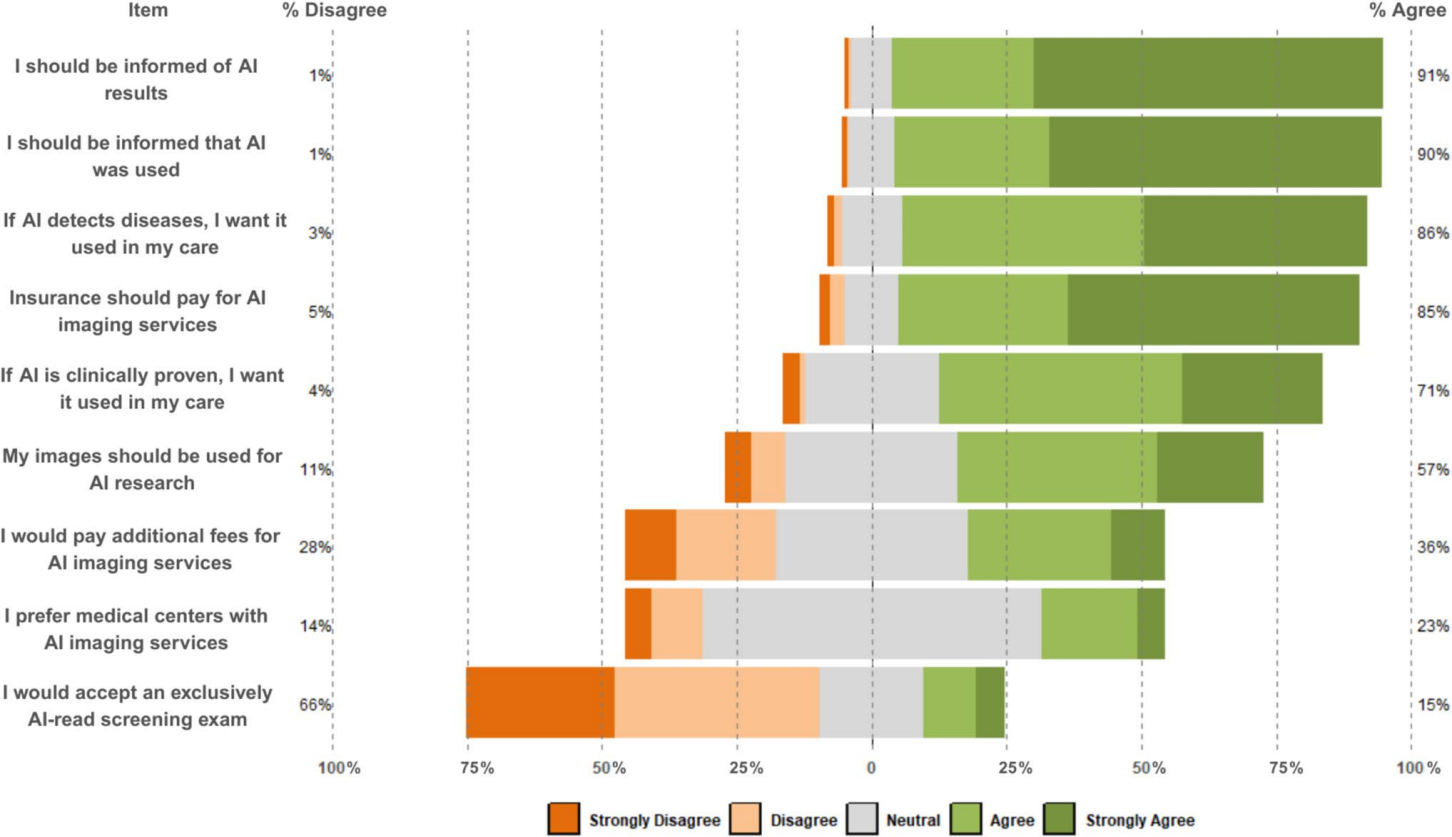
Likert plot of participants’ responses to the study survey’s Likert-scale questions. Descriptions of each item’s content and the item number in parentheses are included for reference

**Fig. 4:**
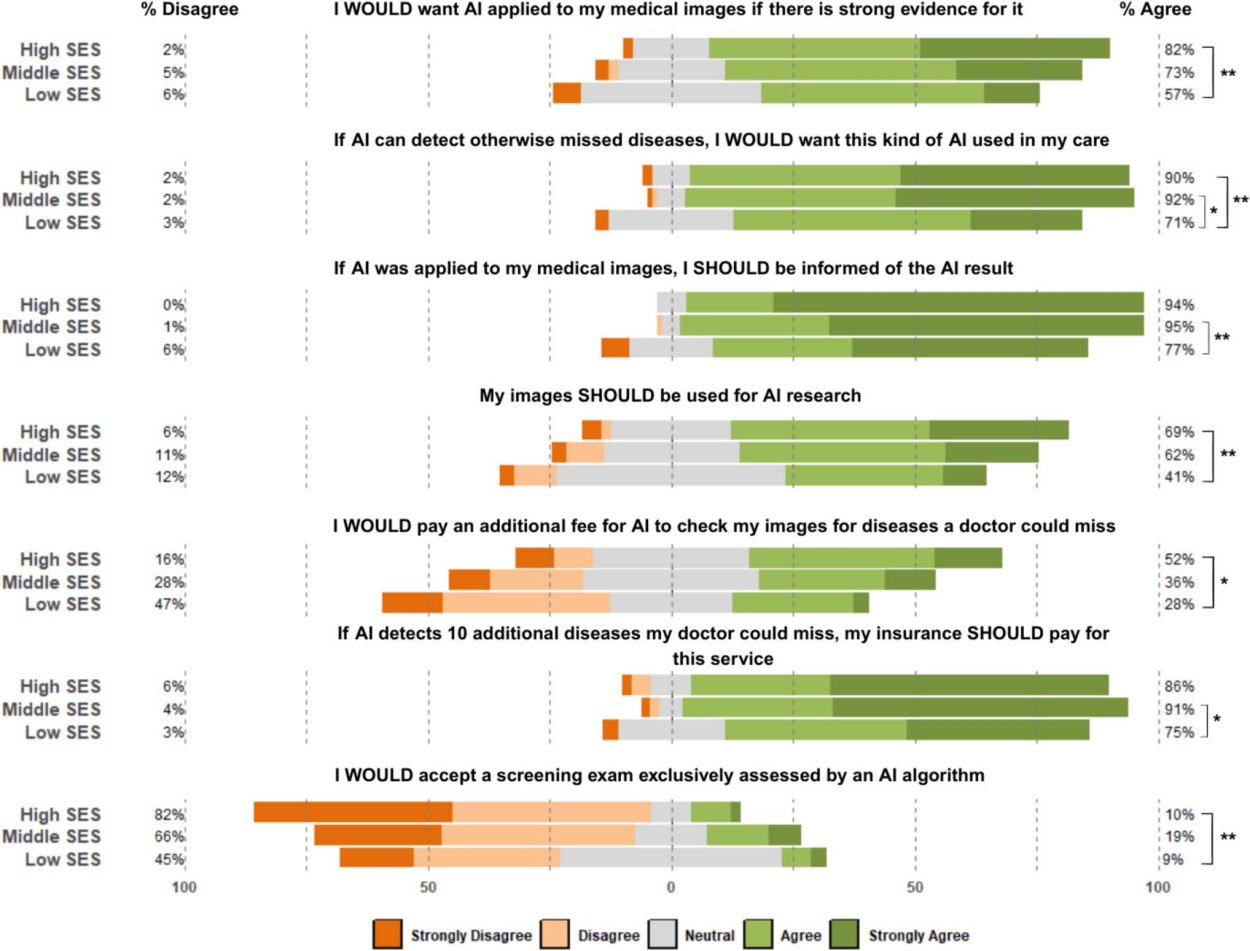
Likert plots depicting differences in patients’ survey responses according to SES (Socioeconomic Status). *p ≤ 0.05; **p ≤ 0.01; ***p ≤ 0.001

A large majority of patients, 89.2% (199/223), expressed that medical centers should inform patients regarding the use of FDA-cleared AI algorithms in clinical care. Concerning the application of AI to medical images, 28.8% (65/226) of patients agreed and 61.5% (139/226) strongly agreed that if AI were applied to their images, they should be informed that AI was used. A full illustration of patients’ responses to the survey’s Likert-scale questions is depicted in Fig. 3. Additionally, 45.1% (102/226) of patients agreed, and 26.1% (59/226) strongly agreed that AI should be applied to medical images if it is proven to improve clinical care. SES was significantly associated with these opinions (Kruskal–Wallis *p* = 0.005), with those in the high SES group indicating a greater preference for AI (82.4% agreeing/strongly agreeing) than individuals in the low SES group (57.1%; Dunn’s *p* = 0.003). For survey items in which SES was significantly associated with participants’ responses, Likert plots depicting differences between SES groups can be found in Fig. 4.

Most of the participants, with 44.9% (101/225) agreeing and 41.3% (93/225) strongly agreeing, expressed a desire for AI use in their care if it can detect diseases in their images that might otherwise be missed, which was also influenced by SES (Kruskal–Wallis *p* = 0.003). While individuals in the high and middle SES groups did not differ in desire for AI (90.2% vs. 92.3% agreeing/strongly agreeing), both endorsed a greater desire for AI than the low SES group (71.4%; Dunn’s *p* = 0.019 and p = 0.002, respectively).

**Fig. 5:**
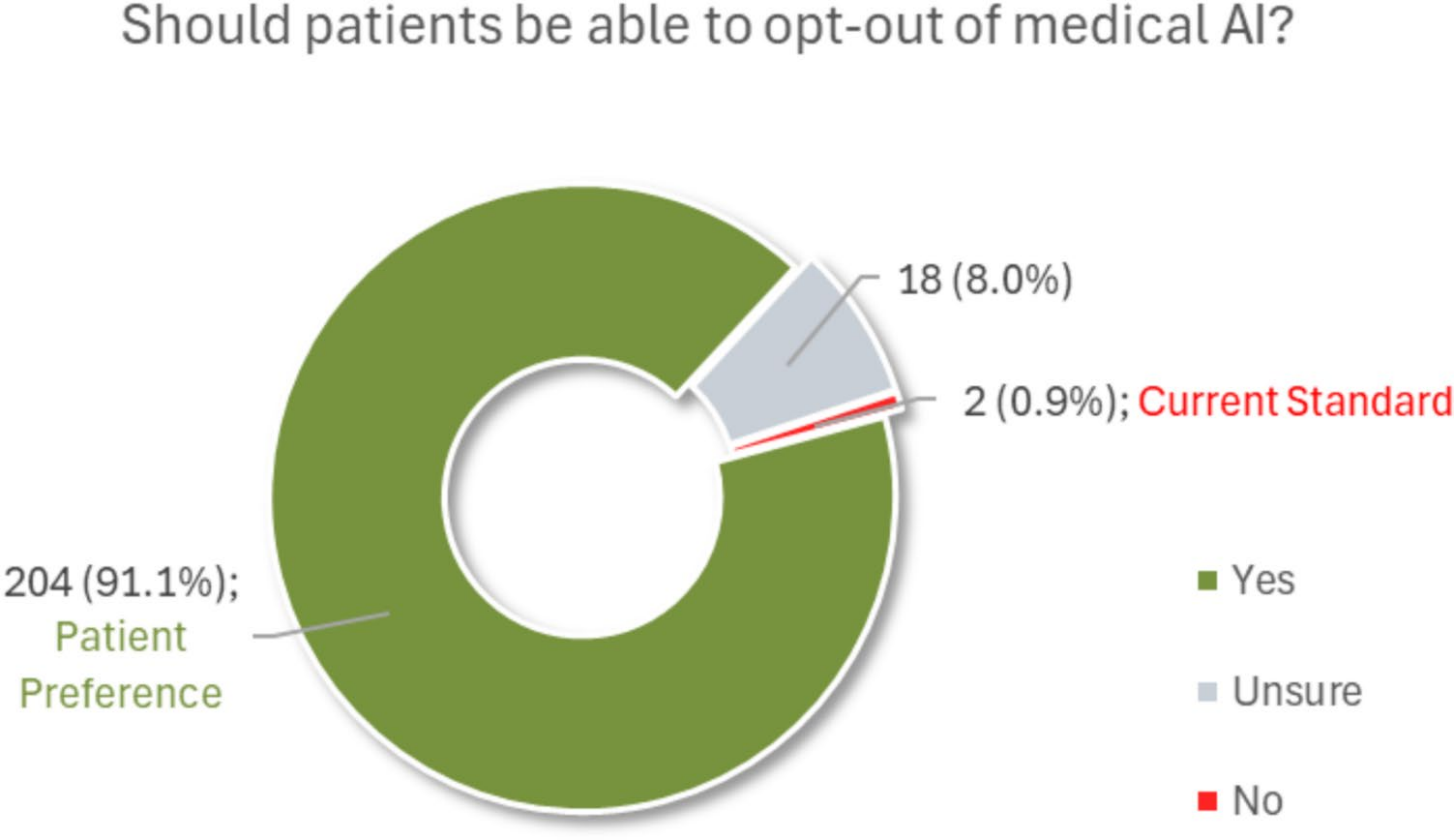
Pie charts of participants’ responses to the question, “Should patients have the right to opt out of using medical AI, even if the medical doctors believe it is helpful?” Study findings (patient preferences) incongruent with most radiology departments’ practices (current standard) regarding artificial intelligence are highlighted in the chart labels

Majority of participants felt that patients should have the right to opt out of using medical AI, even if the medical doctors believe it is helpful, where 91.1% (204/224) designated “Yes…” while only 8.9% (20/224) expressed that they were unsure or did not believe patients deserved the right. Participant responses to this item are depicted in Fig. 5.

A majority of patients supported the use of their radiology images for AI research at academic medical centers, with 19.8% (44/222) strongly agreeing, 36.9% (82/222) agreeing, and 32.0% (71/222) remaining neutral on this statement. SES was significantly associated with these responses (Kruskal–Wallis *p* = 0.022), with high SES individuals agreeing more to the use of their images for AI research (69.4% agreeing/strongly agreeing) than their low SES counterparts (41.1%; Dunn’s *p* = 0.018).

The seven Likert-scale questions assessing patient attitudes toward AI use in medical imaging (all Likert-scale items except the two regarding patient expectations for being informed of AI use) demonstrated good internal consistency, with a Cronbach’s alpha of 0.77 (95% CI: 0.72, 0.82).

### Patient Perspectives on Receiving AI Results

**Fig. 6:**
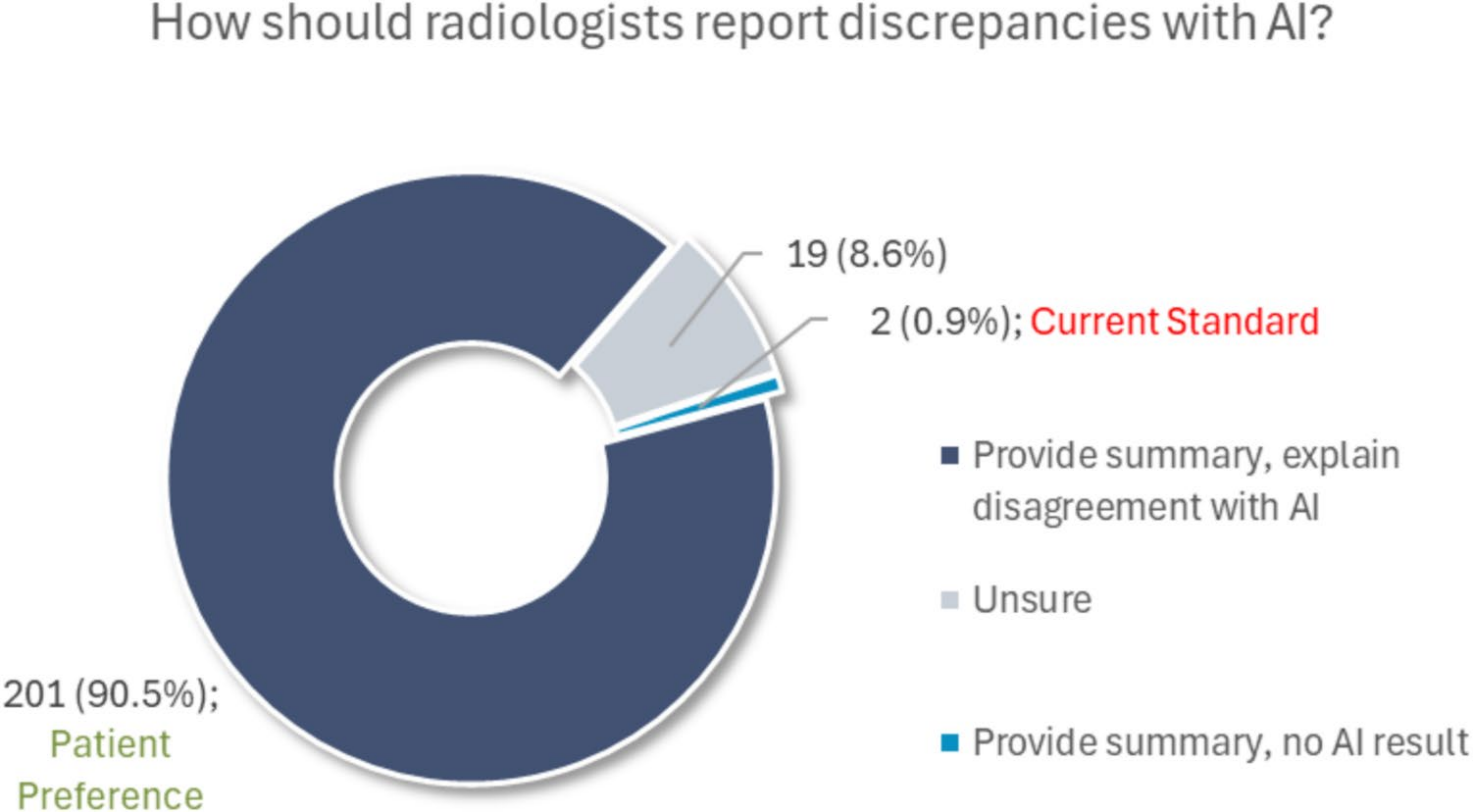
Pie charts of participants’ responses to the question, “If a doctor used AI to assist with the interpretation of your medical images and the doctor disagrees with the AI result, how should the doctor inform the patient in their report?” Study findings (patient preferences) incongruent with most radiology departments’ practices (current standard) regarding artificial intelligence are highlighted in the chart labels

A majority of participants indicated that they should be informed of AI results when used in the interpretation of their medical imaging, with 26.3% (59/224) agreeing and 64.7% (145/224) strongly agreeing. Participants’ responses were significantly associated with their SES (Kruskal–Wallis *p* = 0.013) as individuals in the middle SES group agreed at a greater rate that AI results should be offered (95.2% agreeing/strongly agreeing) compared to individuals in the low SES group (77.1%; Dunn’s *p* = 0.011). Further, when AI is used to assist radiologists in interpreting medical images, 87.9% (196/223) of respondents believed that disclosure of AI involvement and clarification of any discrepancy should be considered standard care. In instances where radiologists’ interpretations differ from AI outputs, 90.5% (201/222) of patients felt that physicians should provide a comprehensive explanation outlining the rationale for their differing conclusions. This preference can be visualized in Fig. 6.

**Fig. 7:**
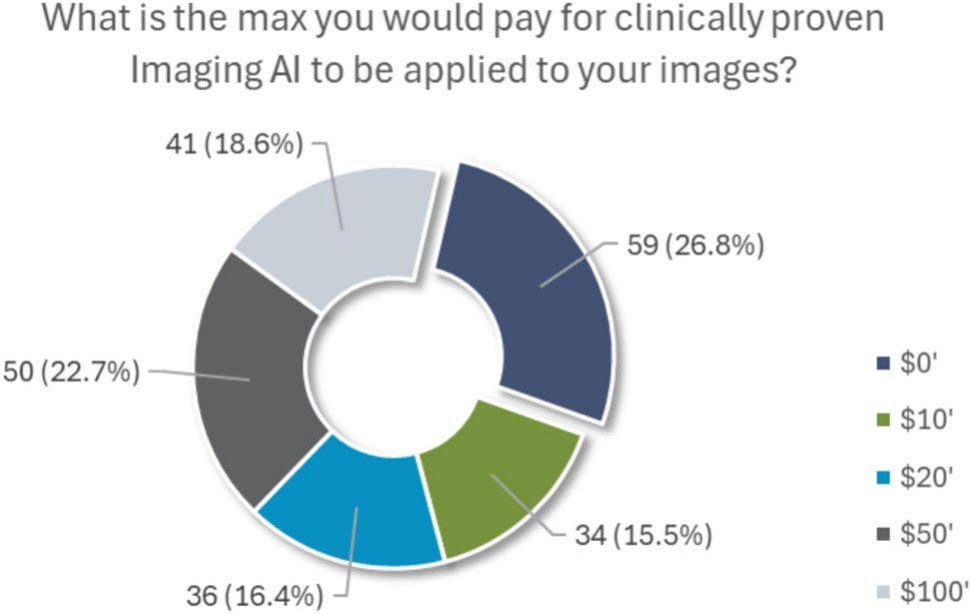
Pie chart of participants’ responses to the question, “If your medical insurance would NOT pay for AI to detect additional serious diseases that your doctor could miss, what is the maximum amount that you would pay out of pocket for this service?”

When asked how radiologists should proceed if they disagree with AI results for medical images, 49.8% (109/219) indicated that doctors should rely on their judgment and the AI results without disclosing the AI findings. Among the 50.2% (110/219) who selected the “Other” option, many respondents provided free-text comments expressing a strong preference to be informed of the AI results, even when these differ from the radiologist’s assessment. In addition, 65.6% (143/218) of participants indicated that they disagree or strongly disagree with accepting a screening imaging exam exclusively interpreted by an AI algorithm, while 15.1% (33/218) agreed or strongly agreed they would be willing to accept such an exam. Participants’ likelihood to accept an exclusively AI-read exam was associated with their SES (Kruskal–Wallis *p* = 0.008), with the high SES group stating that they would not accept the exam at a greater frequency (81.6% disagreeing/strongly disagreeing) than the low SES group (45.5%; Dunn’s *p* = 0.008) (Fig. 7).

### Patient Perspectives on the Economics of AI

A minority of participants expressed willingness to pay an additional fee for AI to review their medical images for diseases that might be missed by a doctor, with 26.6% (59/222) agreeing and 9.9% (22/222) strongly agreeing. This willingness to pay was associated with SES (Kruskal–Wallis *p* = 0.015), as individuals in the high SES group showed more willingness to pay for this AI service (52.0% agreeing/strongly agreeing) than those in the low SES group (28.1%; Dunn’s *p* = 0.012). In contrast, many patients felt that such AI services should be covered by medical insurance to detect conditions that may not be detected; 31.2% (68/218) agreed and 54.1% (118/218) strongly agreed. Patient beliefs on insurance’s obligation to pay were associated with their SES (Kruskal–Wallis *p* = 0.020), with the middle SES group agreeing that insurance should cover this service (91.3% agreeing/strongly agreeing) more than the low SES group (75.0%; Dunn’s *p* = 0.016). When asked about paying out-of-pocket if insurance did not cover the cost, 57.3% (126/220) of participants reported they would be willing to pay between $20 and $100, while 26.8% (59/220) stated that they would not pay any additional fee. Patients’ inclination to pay a higher amount for AI services was associated with their SES (Chi-square *p* = 0.002), as both the high SES and middle SES groups indicated that they would pay more for the service (56.0% and 44.7% willing to pay > $20, respectively) than those in the low SES group (14.7%; Fisher’s exact *p* < 0.001 and *p* = 0.034, respectively).

## Discussion

This single-center cross-sectional survey of participants undergoing imaging exams assessed patient self-perceived knowledge and perspectives regarding the use of AI in medical imaging, with particular attention to the influence of socioeconomic status. Our study found that most patients reported minimal to no knowledge of AI in medicine, with higher-SES individuals reporting greater familiarity. A large majority believed that medical centers should inform patients when FDA-cleared AI algorithms are used in clinical care. This is different from current practice, where AI usage in healthcare facilities is largely undisclosed. While half of the participants were neutral about attending AI-integrated healthcare facilities, many expressed a preference for AI use if it could help detect diseases that might otherwise be missed. Additionally, most participants wanted the option to opt out of AI involvement in their care.

Our findings are consistent with recent studies examining patient perspectives on AI in healthcare (not specifically medical imaging), both within the U.S. and internationally, particularly in demonstrating strong patient preferences for transparency regarding the AI use without outright opposition to its implementation [3, 5–7]. Khullar et al. similarly reported that most patients valued being informed about the role of AI in their medical care [3]. Despite this growing interest in transparency, AI use in clinical practice remains largely undisclosed. To the best of our knowledge, at our institution, and likely at many others, patients are not informed when AI is involved in their care. Although several AI applications are currently in use within our medical imaging department, their use is neither documented in the electronic medical record nor communicated to patients.

Busch et al. conducted a survey across 43 countries and found that most patients preferred visiting healthcare facilities that use AI. In contrast, only about a fifth of patients in our study expressed this preference, while nearly 50% selected a “neutral” option—an option not available in the Busch et al. study. Our findings suggest that patients may not actively prefer imaging centers that use AI. We posit this may be due to limited knowledge about AI, lack of transparency or understanding in how it is applied in their care, and the direct benefits received from the technology.

These findings have important implications for clinical practice. As AI tools become increasingly integrated into the diagnostic and decision-making workflow, radiology practices must consider how to address the patients’ expectations for transparency and informed consent. The lack of documentation and disclosure not only challenges ethical standards for patient autonomy but may also erode trust if patients later discover AI was used without their knowledge. Establishing standardized practices for documenting AI use and developing clear communication strategies to explain AI involvement are needed. A simple approach may be to provide patients with a reference document outlining the AI policies and applications in use at the practice, similar to the Notice of Privacy Practices required under HIPAA. This would provide transparency without being overly burdensome or theoretically increase medicolegal risk, which is beyond the scope of this investigation.

In addition to transparency, many participants in our study expressed a desire to have the option to opt out of AI use in their care, an observation consistent with findings by Richardson et al., who similarly reported patient interest in the ability to decline medical AI involvement. Although preferred by patients, accommodating individual patient preferences for AI use in radiology may not be pragmatic and could have unintended consequences, especially if radiologists become dependent or expect certain functionality in their diagnostic workflows [5]. To illustrate this further, patients do not select the technical parameters of an imaging exam, such as the use of filtered back projection, iterative reconstruction, or deep learning reconstruction, and, similarly, are not expected to dictate whether AI is used in image interpretation when its use falls within the scope of standard radiologic practice. When AI is applied as a tool to assist the radiologist, rather than operating autonomously, its use aligns with established clinical workflows and professional responsibilities. In contrast, fully autonomous AI interpretation without radiologist oversight was opposed by approximately 66% (143/218) of participants in our study, suggesting that informed consent would be warranted in such cases. Importantly, a distinction must be made between patient autonomy (the right of individuals to make informed decisions about their care) and radiologist autonomy, wherein patients rely on the expertise of radiologists to ensure that diagnostic exams are conducted and interpreted using the most accurate and appropriate methods available.

Patient willingness to share imaging data for AI research profoundly impacts medical imaging innovation, as restricted access could limit algorithm development, introduce bias, and impede advances that contribute to generalizable knowledge that benefit all patients. Our finding that 11.2% (25/222) of participants disagreed with using their radiology images for academic AI research requires additional context. Our survey did not specify whether images would be de-identified, require explicit consent, or distinguish academic from commercial applications. De-identified protected health information may not require individual permission for research, and institutional review boards may waive consent for retrospective studies using de-identified data presenting minimal risk [30]. Previous studies have demonstrated that patients generally support sharing de-identified data for research benefiting the common good but may prefer not to have their data shared for commercial use cases [31]. The lack of these distinctions may have influenced participant responses and are important considerations for future research on patient data-sharing preferences and policy.

Our study suggests that SES plays a role in shaping patient preferences toward AI in medical imaging. Although a comprehensive explanation for the tendency of individuals with lower SES to be less supportive of medical AI compared to their higher-income counterparts is likely multifactorial, economic data may offer a piece of the puzzle. A 2024 report from the Heldrich Center for Workplace Development found that those earning under $50,000 express more concern about AI-driven job displacement [32]. Moreover, this demographic expressed a greater desire for governmental intervention to mitigate AI’s economic impact than individuals with incomes exceeding $100,000 annually. Other recent studies have described a potential relationship between fear of job loss and distrust in AI [33]. Socioeconomic concerns likely shape AI acceptance beyond the workplace, influencing healthcare perspectives as well. Our study also finds that individuals with lower SES report less knowledge about AI than those with higher SES. This disparity in knowledge can impact attitudes and behaviors, similar to observations during the COVID-19 pandemic, where insufficient knowledge about vaccines correlated with higher vaccine hesitancy [34, 35]. However, it is important to note that our measure of knowledge reflects self-perceived understanding rather than objective knowledge of AI, and such self-perceptions may be influenced by confidence levels that vary systematically across socioeconomic groups.

These findings are particularly important given that individuals with lower SES are disproportionately more likely to utilize emergency department services [36, 37] and may more frequently present with acute conditions that are well-suited for AI-based triage systems [38–40]. If patients from lower SES groups elect to opt out of AI-assisted triage at higher rates than their higher SES counterparts, significant clinical consequences could ensue. Without the support of AI, cases with critical findings may be undertriaged, leading to radiologists perceiving them as less urgent or biasing the radiologist into the absence of a critical finding, potentially resulting in delayed interpretation when timely reads are essential or increased error rates, which could exacerbate existing healthcare disparities [41, 42].

Furthermore, our study also found that individuals with higher SES are more willing to invest in clinically validated AI services. In a pay-to-play environment, this willingness could result in AI tools disproportionately benefiting those with higher SES, further widening disparities in healthcare access and outcomes. These considerations highlight the critical need not only for improved patient education and engagement regarding AI in medical imaging, but also for policy interventions that ensure coverage and financial accessibility of beneficial AI tools for all patients, regardless of their ability to pay. Without such coverage mechanisms, even well-informed patients from lower socioeconomic backgrounds may be unable to benefit from these technological advances.

Our study has several limitations. First, this study was conducted at a single center within a specific geographic region of the United States and limited to an outpatient imaging setting; therefore, the findings may not be generalizable to other regions or healthcare environments. Second, the survey instrument was not pilot tested or formally validated prior to implementation, and some question wording may have introduced bias (e.g., using “hide” rather than more neutral language such as “not discuss” when referring to AI results), which could have influenced participant responses. Additionally, the survey did not provide detailed descriptions of specific medical imaging AI applications, which may have influenced patients’ understanding and responses. Further research is needed to explore how patients perceive and differentiate among various types of AI applications in medical imaging. Third, *p*-values were not adjusted for multiple comparisons when examining associations between SES and patients’ responses as this study was exploratory in nature, though we did apply appropriate corrections for the post-hoc tests comparing different SES levels; this approach increases the risk of type I error but allows for the identification of potential relationships that can be investigated more rigorously in future studies. Lastly, we did not track individuals who declined participation, preventing calculation of a response rate and assessment of non-response bias. This could introduce survey participation bias, as survey completers may have had stronger opinions about AI in radiology than non-participants. For example, our sample showed an overrepresentation of female participants (63.3%) compared to the female gender distribution of patients receiving imaging at our facility (56.1%). Without tracking who declined participation, we cannot determine whether this reflects differential approach rates, consent rates by gender, or both. Future studies should document both the number approached and reasons for non-participation to better evaluate sample representativeness. Despite these limitations, the study has several notable strengths. It includes a large and demographically diverse sample, stratified across key variables such as age, sex, race, and socioeconomic status, which enhances the relevance and applicability of the findings. Additionally, the survey addressed a broad range of questions related to medical AI, offering a comprehensive view of patient attitudes and preferences that adds meaningful depth to the current literature.

To our knowledge, our study is the first to systematically examine patients’ perspectives on key aspects of AI integration in radiology, including their opinions on the right to opt out of an AI-assisted workflow, their expectations for how physicians should handle disagreements with AI-generated findings, and their preferences regarding whether and how AI-derived results should be communicated to them. In conclusion, our study underscores the need for greater transparency and communication with patients regarding the use of AI in medical imaging and highlights the discrepancy between patient preferences and current clinical practice.

## Data Availability

A.D.S. is a co-owner of AI Metrics, a co-owner of Body Check LLC, and the owner of Radiostics. A.D.S. also holds multiple patents issued and pending related to image processing algorithms. These relationships had no influence on the design, conduct, or reporting of this study.

## Declarations

S.A.R. career development scholarship from the American Roentgen Ray Society, consulting fees from Radiostics, Bunkerhill Health, Innolitics, and TeraRecon, salary support Coreline Soft, stock options Bunkerhill Health; co-owner Body Check LLC, multiple patents issued and pending on image processing algorithms. A.D.S. co-owner AI Metrics, co-owner, Body Check LLC, owner Radiostics, multiple patents issued and pending on image processing algorithms.
